# Vector flow mapping analysis of left ventricular vortex performance in type 2 diabetic patients with early chronic kidney disease

**DOI:** 10.1186/s12872-023-03474-7

**Published:** 2023-09-01

**Authors:** Xiaoxue Chen, Fang Qiu, Wei Wang, Zhengqin Qi, Damin Lyu, Kun Xue, Lijuan Sun, Degang Song

**Affiliations:** 1 Department of Ultrasound, First Hospital of Qinhuangdao, Hebei Medical University, No.258, Wenhua Road, Qinhuangdao, 066000 Hebei China; 2 Hebei Key Laboratory of Vascular Homeostasis and Hebei Collaborative Innovation Center for Cardio- cerebrovascular Disease, No. 215, Hepingxi Road, Shijiazhuang, 050000 Hebei China; 3 Department of cardiology, First Hospital of Qinhuangdao, Hebei Medical University, No.258, Wenhua Road, Qinhuangdao, 066000 Hebei China; 4 Department of Cardiac Ultrasound, Second Hospital of Hebei Medical University, 215 Hepingxi Road, Shijiazhuang, 050000 Hebei China; 5 Department of neurology, First Hospital of Qinhuangdao, Hebei Medical University, No.258, Wenhua Road, Qinhuangdao, 066000 Hebei China

**Keywords:** Left ventricular vortex, Vector flow mapping, Chronic kidney disease, Diastolic dysfunction, Glycemia, Early stages

## Abstract

**Background:**

Diabetes is the leading cause of chronic kidney disease (CKD) and contributes to an elevated incidence of diastolic dysfunction in the early stages of CKD. Intracardiac vortex is a novel hemodynamic index for perceiving cardiac status. Here, we visualized left ventricular (LV) vortex characteristics using vector flow mapping (VFM) in type 2 diabetic patients with early CKD.

**Methods:**

This cross-sectional study included 67 controls and 89 type 2 diabetic patients with stages 2-3a CKD. All subjects underwent transthoracic echocardiographic examination. LV anterior vortex during early diastole (E-vortex), atrial contraction (A-vortex) and systole (S-vortex) were assessed using VFM in the apical long-axis view. Its relation to glycemia or LV filling echocardiographic parameters were further analyzed using correlation analysis.

**Results:**

Type 2 diabetic patients with early CKD had a small area (439.94 ± 132.37 mm^2^ vs. 381.66 ± 136.85 mm^2^, *P* = 0.008) and weak circulation (0.0226 ± 0.0079 m^2^/s vs. 0.0195 ± 0.0070 m^2^/s, *P* = 0.013) of E-vortex, but a large area (281.52 ± 137.27 mm^2^ vs. 514.83 ± 160.33 mm^2^, *P* ˂ 0.001) and intense circulation (0.0149 ± 0.0069 m^2^/s vs. 0.0250 ± 0.0067 m^2^/s, *P* < 0.001) of A-vortex compared to controls. CKD patients with poorly controlled hyperglycemia had stronger A-vortex (area: 479.06 ± 146.78 mm^2^ vs. 559.96 ± 159.27 mm^2^, *P* = 0.015; circulation: 0.0221 ± 0.0058 m^2^/s vs. 0.0275 ± 0.0064 m^2^/s, *P* < 0.001) and S-vortex (area: 524.21 ± 165.52 mm^2^ vs. 607.87 ± 185.33 mm^2^, *P* = 0.029; circulation: 0.0174 ± 0.0072 m^2^/s vs. 0.0213 ± 0.0074 m^2^/s, *P* = 0.015), and a longer relative duration of S-vortex (0.7436 ± 0.0772 vs. 0.7845 ± 0.0752, *P* = 0.013) than those who had well-controlled hyperglycemia. Glycemia, and E/A (a LV filling parameter) were respectively found to had close correlation to the features of A-vortex and S-vortex (all *P* < 0.05).

**Conclusions:**

Abnormal LV vortices were detected in type 2 diabetic patients with early CKD using VFM, especially in those who neglected hyperglycemic control. LV vortex might be a promising parameter to slow or halt the hyperglycemia-induced diastolic dysfunction in early CKD.

**Supplementary Information:**

The online version contains supplementary material available at 10.1186/s12872-023-03474-7.

## Background

Type 2 diabetes is the most common cause of chronic kidney disease (CKD) worldwide, and carries with it a severe public health burden [1, 2]. Type 2 diabetic CKD induces significant abnormalities of cardiac anatomy and functions [3]. Thus, the cardiovascular morbidity in those patients is substantially high, even in the early stages [4, 5]. Myocardial stiffening due to hyperglycemia and hyperfiltration already appears and advances in early CKD, resulting in imperceptible subclinical diastolic dysfunction of heart without alteration in morphology [6–8]. These observations underscore the necessity for assessing and managing the subclinical diastolic dysfunction of type 2 diabetic CKD patients in the early stages, when the best possibility of effective treatment exists.

Thanks to the rapid development of novel cardiac image techniques, increasing attentions have been recurrently dedicated to assessing cardiac flow patterns in multiple pathological conditions, with vortices playing an essential role among them, contribute to proper cardiac function [9, 10]. Cardiac vortex has been considered to play a critical role in avoiding energy loss that would appear in a chaotic distribution of cardiac blood flow [11, 12]. Additionally, vortex seems to be involved in atrioventricular coupling and redirection of left ventricle (LV) inflow toward outflow tracts [13]. Vector flow mapping (VFM) has positive efficacy in visualization and quantification of vortices appearing in cardiac chambers, with a suitable clinical application owing to its convenient and non-invasive characteristics [14]. The clinical value of intracardiac vortex was initially suggested in 2006, Gharib et al. showed that diastolic dysfunction can be “uniquely and sensitively” reflected by the early diastolic vortex formation [15]. Following this direction, a study including 62 patients with diastolic dysfunction concluded that cardiac vortices can be efficiently used to evaluate the grades of diastolic dysfunction [16]. Mounting studies suggested the diagnostic and prognostic significance of the VFM-visualized vortices in cardiac functions [17–19], cardiac resynchronization therapy [20], coronary atherosclerotic heart disease [21] and heart valve disease [22, 23].

Vortex has been confirmed to provide intracardiac dynamic information in patients with Uremia (late stages CKD) using VFM and it might be a valuable supplement for assessing LV function [24]. However, the vortex behavior and its potential clinical value in early CKD patients remain unclear. In the absence of cardiac morphological changes, it has been demonstrated that hemodynamic abnormalities due to the subclinical diastolic dysfunction can exist in the heart of early CKD [7, 25]. Our work aimed to investigate the dynamic changes of LV vortex behavior using VFM in the type 2 diabetic patients with early CKD and to testify whether vortex is a potential way for observing the status of intracardiac blood flow and is useful for evaluating the diastolic dysfunction of those patients. Hyperglycemia, as the core clinical management target, has been considered to aggravate the progression of cardiac dysfunction in patients with diabetes and CKD [26]. Therefore, we further explored the effect of hyperglycemia on the cardiac vortex behavior in those patients. This study was expected to provide valuable clinical data on the diagnostic and prognostic significance of LV vortices in the type 2 diabetic patients with early CKD.

## Methods

### Study population

Eighty-nine type 2 diabetic patients with early CKD in the First Hospital of Qinhuangdao and the Second Hospital of Hebei Medical University from 2021 to 2022 were recruited to participate this cross-sectional study. The participants were male or female, 30–80 years of age, type 2 diabetes with CKD stages 2-3a (estimated glomerular filtration rate 50–80 ml/min/1.73 m^2^), and had an established diagnosis of diabetic nephropathy as the underlying cause of renal disease. Exclusion criteria included inadequate echocardiographic visualization, non-diabetic CKD, essential hypertension, heart failure, any type of valvular heart disease, congenital heart disease, hypertrophic or restrictive cardiomyopathies, myocarditis, pericarditis, arrhythmia, tachycardia, or an unstable clinical or hemodynamic profile. Glycated hemoglobin (HbA1c) is a clinical parameter representing glycemia status. To investigate the effect of hyperglycemia on the LV vortex features in early CKD, the type 2 diabetic patients with early CKD were further divided into well-controlled hyperglycemia (W-HG, HbA1c ˂ 8.0% n = 41) and poorly controlled hyperglycemia (P-HG, HbA1c ≥ 8.0%, n = 48) subgroups according to the recommendation for glycemic control in 2020 KDIGO clinical practice guideline [2]. Sixty-seven age- and sex-matched healthy subjects with no previous history of cardiovascular disease were enrolled as a control group.

The study was approved by the local medical Ethics Committee, and written informed consent was obtained from all participants. All procedures were in accordance with the 1964 Helsinki declaration and its later amendments or comparable ethical standards.

### Transthoracic echocardiography

Two-dimensional and Doppler transthoracic echocardiography were satisfactorily performed by experienced sonographers in all subjects using a Pro-Sound F75 ultrasound device (Hitachi-Aloka Medical Ltd., Tokyo, Japan) with a UST-52105 probe (1–5 MHz). LV end-diastolic diameter (LVEDd) and LV end-systole diameter (LVEDs), were measured using M-mode in the parasternal long-axis view. Then, LV end-diastolic volume (LVEDV), LV end-systolic volume (LVESV), and LV ejection fraction (LVEF) were automatically calculated according to the biplane Simpson’s method [27]. The early (E) and late (A) diastolic mitral inflow velocities, the early (e’) diastolic medial mitral annular velocities were measured in the apical four-chamber view according to the recommendations by the American Society of Echocardiography [27].

### Vector flow mapping analysis

Color Doppler acquisitions were performed from the apical long-axis view in VFM mode, with the Nyquist limit enhanced to mitigate aliasing phenomenon, while maintaining a frame rate > 23 frames/s and a sufficient region of interest to include the entire left ventricle [7]. The data (three cardiac cycles for each subject) were transferred to an offline workstation and for analyzed using VFM analysis software (DAS-RS1, Hitachi Aloka Medical Ltd.) [28]. Intraventricular blood flow was visualized by VFM using velocity vectors based on color Doppler imaging and two-dimensional speckle tracking [13].

Intracardiac swirling flow can be effectively detected using VFM, LV vortices can be visually and quantitatively depicted through 2D streamline maps and vortex maps obtained from VFM processing. Vortex is defined as blood flow that circulates back to its starting point, while flow that does not return to the starting point is not considered a vortex. The software employed in VFM analysis can determine the boundary between vortex and non-vortex regions. VFM enables the detection of the outermost boundary of the vortex, facilitating the quantification of vortex area. The analysis software automatically tracks and displays the vortex area and circulation (total vorticity). Circulation is calculated as the integral of the normal component of “vorticity” (ω) on an arbitrary plane (S) enclosed by a closed curve using the following formula:

1 $$Circulation = \iint\limits_S {{w_n}dS}$$

It is calculated by accumulating the velocity component of a tangent on a closed curve, so the circulation becomes higher when the flow velocity is higher [29]. The area and circulation of the LV largest clockwise vortex during systole (S-vortex), early diastole at the time of E wave (E-vortex), and late diastole at the time of A wave (A-vortex) were recorded as previously described [28]. Additionally, Relative duration of diastolic vortex and Relative duration of systolic vortex were measured according to the previous work [13], and respectively calculated as the “total duration of E-vortex and A-vortex/duration of diastole phase in a cardiac cycle” ratio, and the “duration of S-vortex /duration of systolic phase in a cardiac cycle” ratio.

### Statistical analysis

All statistical data were analyzed using SPSS 21.0 (SPSS, Inc., Chicago, IL). Normality was estimated by the Kolmogorov-Smirnov test. Continuous data with normal distributions were expressed as the mean ± Standard Deviation (SD), and those with skewed distributions were presented as median and inter-quartile ranges. Comparisons between two groups were evaluated using Student’s t-test (nondirectional). Spearman’s rank correlation test was employed to investigate the relationships between vortex profiles and some of the echocardiographic measurements of subclinical LV dysfunction.

To assess intra-observer and interobserver variabilities, the vortex parameters for approximately 15% of all of the participants randomly selected were repeated on two different occasions and independently performed by a second examiner on the same day, in a manner that was blinded to the previous measurements. Agreement between the repeated measurements was determined using Bland–Altman analyses [30].

## Results

### Baseline clinical and echocardiographic characteristics

Demographics, baseline clinical characteristics, and echocardiographic findings were summarized in Table 1. There were no differences in demographics between the control group and the type 2 diabetic CKD group. In clinical characteristics, type 2 diabetic patients with early CKD exhibited high levels of HbA1c, Triglycerides, and LDL, while they had low levels of eGFR, ALB, and hemoglobin compared to the healthy participants. Moreover, our echocardiographic findings revealed that type 2 diabetic CKD patients had similar LVEDD/ESD, LVEDV/ESV, LVEF, and E wave to the healthy subjects, but enhanced E/A ratio and A. In the present study, we attached importance to the influence of blood glucose on the LV vortex in early CKD. Therefore, the type 2 diabetic CKD patients were further divided into W-HG and P-HG subgroups. Except for the HbA1c level, no significant differences were found in demographics and baseline clinical characteristics between the two subgroups. Consistently, there were no significant differences in conventional 2D parameters. The patients in the P-HG subgroup had similar LVEDD/ESD, LVEDV/ESV, and LVEF to the patients in the W-HG subgroup. However, we found a different echocardiographic feature in the Doppler-related parameters between the two subgroups. The type 2 diabetic CKD patients with poorly controlled hyperglycemia had higher A, and E/A than those with well-controlled hyperglycemia.

**Table 1:** Baseline clinical and echocardiographic characteristic

Parameters	Control	Type 2 diabetic patients with early CKD		
		All	W-HG	P-HG
	n = 67	n = 89	n = 41	n = 48
Age (y)	44.72 ± 12.93	45.56 ± 11.27	43.17 ± 13.69	47.77 ± 12.43
Sex (male/female)	36/31	48/41	22/19	26/22
BSA (m^2^)	1.61 ± 0.10	1.64 ± 0.11	1.63 ± 0.13	1.65 ± 0.12
HR (beat/min)	71.61 ± 12.23	72.76 ± 11.73	72.21 ± 11.94	73.27 ± 12.35
eGFR (ml/min/1.73 m^2^)	101.74 ± 8.45	67.45 ± 8.34*	65.31 ± 10.03	69.37 ± 8. 94
CKD duration (m)	--	30.12 ± 7.17	28.53 ± 8.52	31.78 ± 7.69
HbA1c (%)	5.04 ± 0.81	7.83 ± 0.74*	6.94 ± 0.85	8.74 ± 0.67^**#**^
DM duration (m)	--	91.58 ± 14.57	87.96 ± 15.75	94.03 ± 17.20
Triglycerides (mmol/L)	1.16 ± 0.37	1.84 ± 0.35*	1.85 ± 0.43	1.83 ± 0.32
LDL (mmol/L)	2.67 ± 0.55	3.42 ± 0.83*	3.17 ± 0.94	3.63 ± 0.67
ALB (g/l)	45.08 ± 2.48	34.09 ± 4.79*	33.34 ± 6.03	34.66 ± 4.66
Hemoglobin (g/l)	138.22 ± 11.01	120.08 ± 10.15*	127.72 ± 14.36	115.12 ± 8.91
LVEDD (mm)	46.74 ± 3.69	48.62 ± 5.34	47.23 ± 4.84	49.88 ± 5.86
LVESD (mm)	26.52 ± 4.17	26.91 ± 3.62	25.79 ± 3.77	27.54 ± 4.22
LVEDV (mL)	104.17 ± 18.73	111.64 ± 20.75	103.15 ± 20.48	119.25 ± 22.02
LVESV (mL)	26.54 ± 5.32	26.86 ± 6.93	26.37 ± 7.17	27.11 ± 5.87
LVEF (%)	66.28 ± 10.34	64.12 ± 9.16	65.09 ± 8.01	63.34 ± 10.21
E (cm/s)	73.64 ± 18.08	65.05 ± 16.66*	64.58 ± 16.94	65.45 ± 16.58
A (cm/s)	67.06 ± 16.90	75.88 ± 15.62*	70.13 ± 14.45	80.79 ± 15.02^**#**^
E/A	1.19 ± 0.46	0.89 ± 0.27*	0.96 ± 0.29	0.83 ± 0.24^**#**^
e′ (cm/s)	8.59 ± 2.32	8.46 ± 2.72*	8.67 ± 1.71	7.40 ± 1.44^**#**^

Date given as mean ± Standard Deviation

BSA: body surface area, HR: heart rate, eGFR: estimated glomerular filtration rate, CKD: chronic kidney disease, HbA1c: glycated hemoglobin, DM; diabetes mellitus, LDL: low-density lipoprotein, ALB: albumin, LVEDD/SD: left ventricular end-diastolic/systolic diameter, LVEDV/SV: left ventricular end-diastolic/systolic volume, LVEF: left ventricular ejection fraction, E: mitral early filling wave peak velocity, A: mitral late filling wave peak velocity, e’: early mitral annular peak velocity on septal side. W-HG subgroup: patient with well-controlled hyperglycemia, P-HG subgroup: patients with poorly controlled hyperglycemia. *P < 0.05 vs. Control; ^#^*P* < 0.05 vs. W-HG)

### Intracardiac vortex flow characteristics in type 2 diabetic early CKD

Different cardiac vortices were consistently observed in left ventricle on both anterior and posterior sides of the mitral valve following each trans-mitral filling wave. During early filling, a larger clockwise vortex was observed close to the LV outflow tract, and a smaller counterclockwise one was detected at the posterior side of the left ventricle. The two vortices form at the time of the E wave near the LV base, grow and migrate towards mid-left ventricle, then fade at the end of the E wave. This larger clockwise vortex has been considered to contribute to maintaining blood flow in motion and LV filling by redirecting blood flow in the outflow tract as well as reducing friction forces. During late filling, vortices form again near the LV base at the time of the A wave, including an anterior larger clockwise vortex and a posterior smaller anti-clockwise vortex. The anterior clockwise vortex appears after the onset of peak A-wave can persist into systole phase, and can migrate to the sub-mitral area, reaching its maximal size in isovolumic contraction phase. The proximity of the mitral annulus to the inferior-lateral wall results in small size and short duration of the posterior vortices, which probably impedes its potential hemodynamic relevance. However, the anterior vortices persist longer during the phases of a cardiac cycle, and have been assumed to contribute to optimizing cardiac function. Therefore, we focused on the anterior vortices and examined the behaviors of S-vortex, E-vortex, and A-vortex in this study.

**Fig. 1:**
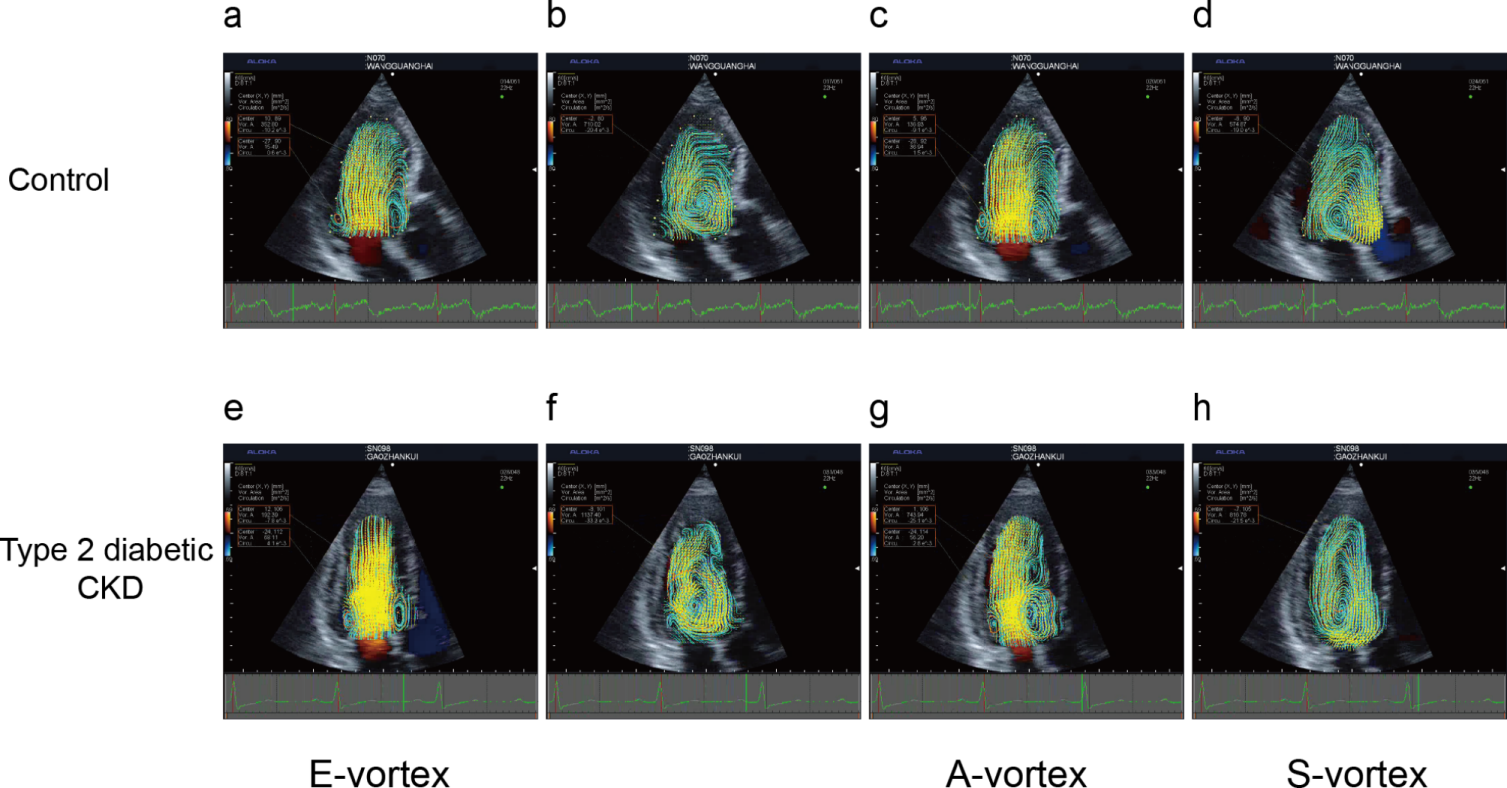
Representative LV vortex images in apical three-chamber view. Representative images of vortex during early diastole **(a)**, vortex at the completion of passive ventricular filling **(b)**, vortex during atrial contraction **(c)** and vortex during systole **(d)** collected from a control participant. Representative images of vortex during early diastole **(e)**, vortex at the completion of passive ventricular filling **(f)**, vortex during atrial contraction **(g)** and vortex during systole **(h)** collected from a type 2 diabetic patient with stages 2-3a

**Fig. 2:**
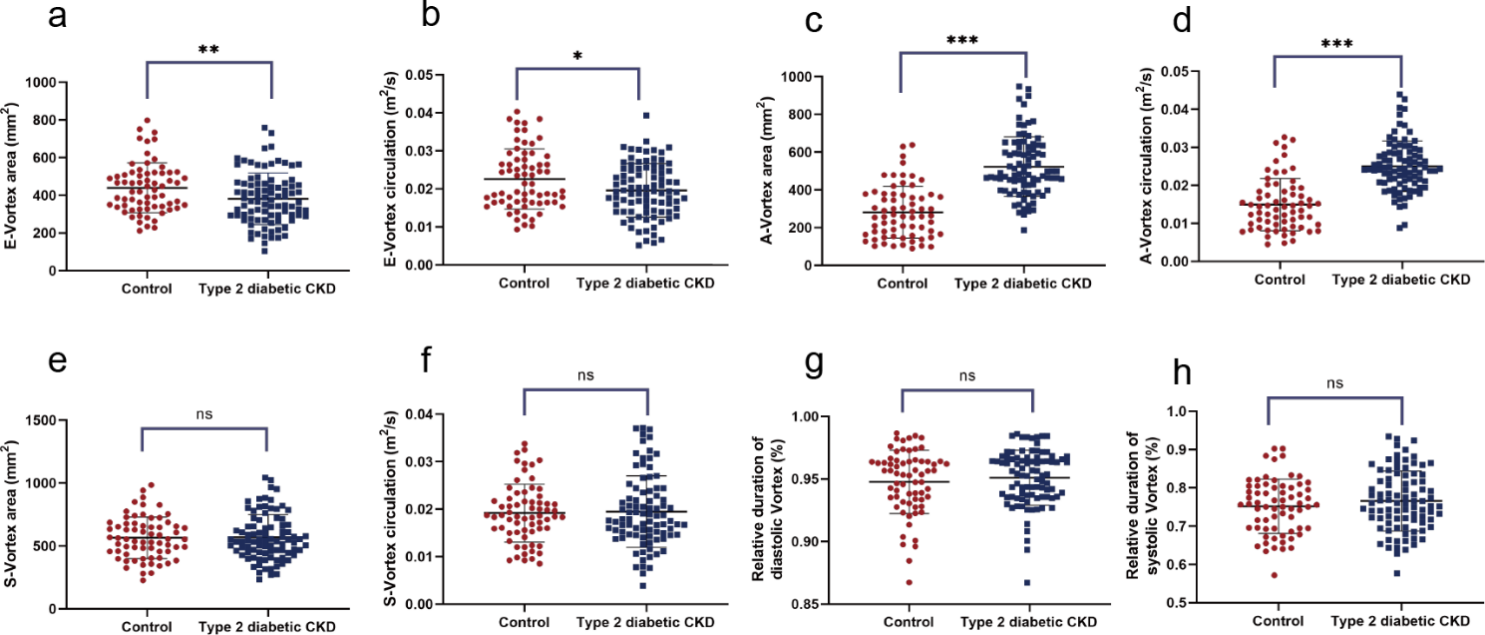
LV vortex features in controls and type 2 diabetic patients with stages 2-3a CKD. **a** E-vortex area. **b** E-vortex circulation. **c** A-vortex area. **d** A-vortex circulation. **e** S-vortex area. **f** S-vortex circulation. **g** Relative duration of diastolic vortex. **h** Relative duration of systolic vortex. Control group: n = 67, type 2 diabetic CKD group: n = 89. All data are expressed as mean ± SD, Student’s t-test * *P* < 0.05, ** *P* < 0.01, *** *P* < 0.001, ns means no statistical significance

E-vortex, A-vortex, and S-vortex in all subjects were satisfactorily visualized on VFM mode in the apical three-chamber view and presented clockwise rotation (Fig. 1). The LV vortex features of all participants were demonstrated in Fig. 2. The E-vortex area of patients with type 2 diabetic early CKD was decreased compared to the healthy controls (439.94 ± 132.37 mm^2^ vs. 381.66 ± 136.85 mm^2^, *P* = 0.008). E-vortex circulation showed a similar tendency to E-vortex area between the control group and the type 2 diabetic CKD group (0.0226 ± 0.0079 m^2^/s vs. 0.0195 ± 0.0070 m^2^/s, *P* = 0.013). Different from the E-vortex, A-vortex presented a strong behavior in size and intensity. Patients with type 2 diabetic early CKD had increased A-vortex area (281.52 ± 137.27 mm^2^ vs. 514.83 ± 160.33 mm^2^, *P* ˂ 0.001) and circulation (0.0149 ± 0.0069 m^2^/s vs. 0.0250 ± 0.0067 m^2^/s, *P* ˂ 0.001) compared to the healthy participants. S-vortex is derived from the A-vortex, and grows into the largest LV vortex during the isovolumetric contraction phase. The S-vortex area (565.23 ± 166.86 mm^2^ vs. 569.33 ± 180.60 mm^2^, *P* = 0.885) and circulation (0.0192 ± 0.0061 m^2^/s vs. 0.0195 ± 0.0075 m^2^/s, *P* = 0.778) were also enhanced in type 2 diabetic CKD group compared to the control, but with no statistically difference. Moreover, we turned to examine the duration of the anterior vortices. The relative duration of diastolic vortex (0.9476 ± 0.0254 vs. 0.9510 ± 0.0220, *P* = 0.376) and relative duration of systolic vortex (0.7517 ± 0.0712 vs. 0.7657 ± 0.0784, *P* = 0.252) respectively showed no statistically significant difference between early CKD patients and healthy controls.

### Hyperglycemia and intracardiac vortex behavior in type 2 diabetic CKD

**Fig. 3:**
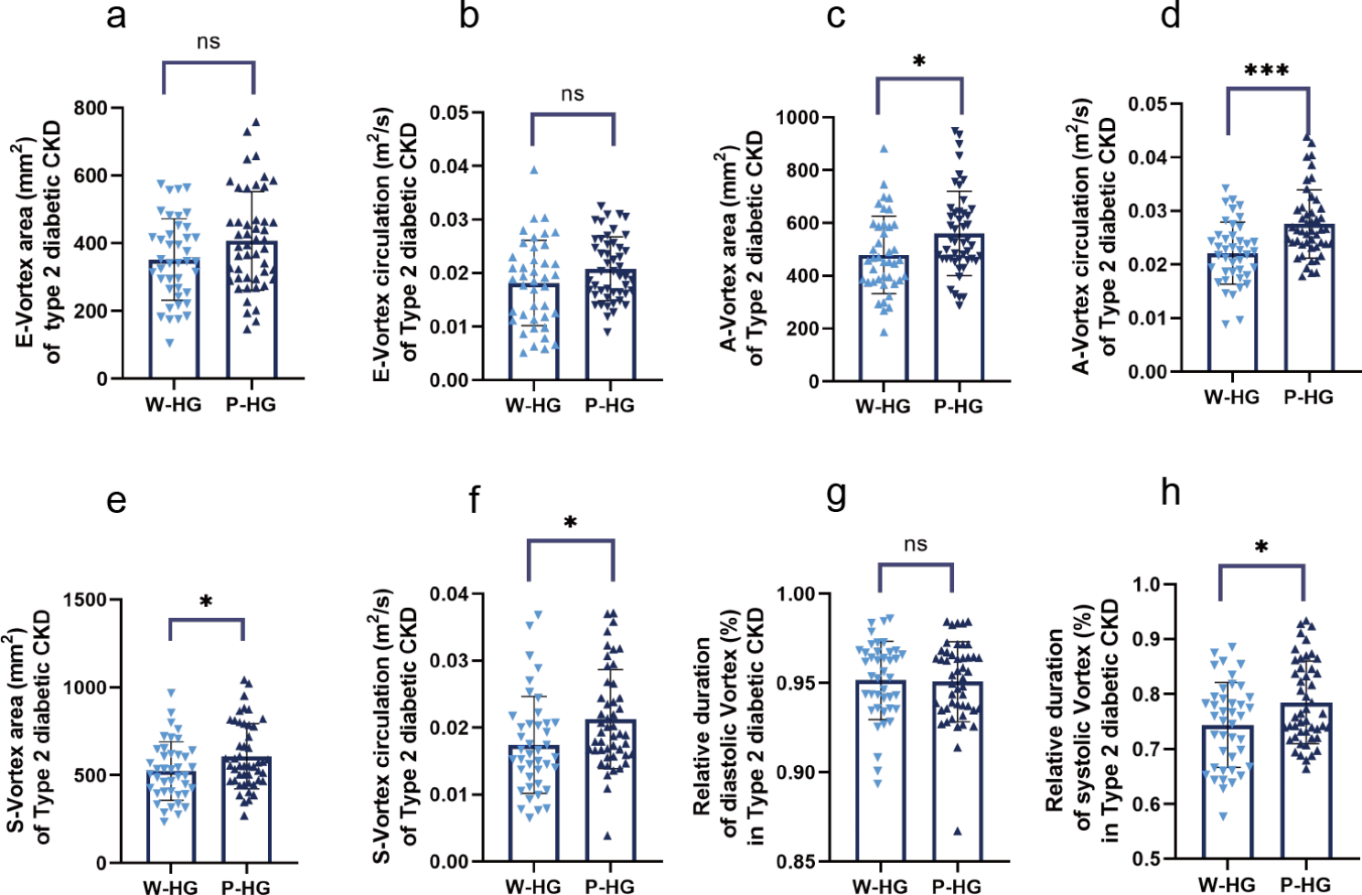
LV vortex features of type 2 diabetic patients with stages 2-3a CKD. **a** E-vortex area. **b** E-vortex circulation. **c** A-vortex area. **d** A-vortex circulation. **e** S-vortex area. **f** S-vortex circulation. **g** Relative duration of diastolic vortex. **h** Relative duration of systolic vortex. W-HG subgroup (n = 41): patient with well-controlled hyperglycemia, P-HG subgroup (n = 48): patients with poorly- controlled hyperglycemia. All data are expressed as mean ± SD, Student’s t-test * *P* < 0.05, *** *P* < 0.001, ns means no statistical significance)

To explore the effect of blood glucose status on intracardiac blood flow of patients with type 2 diabetic early CKD, E-vortex, A-vortex and S-vortex were further investigated between the W-HG subgroup and the P-HG subgroup (Fig. 3). Although the E-vortex exhibited a weak behavior in the type 2 diabetic CKD patients with poorly-controlled hyperglycemia, there was no statistical significance of E-vortex area (351.68 ± 120.84 mm^2^ vs. 407.25 ± 145.54 mm^2^, *P* = 0.056) and E-vortex circulation (0.0181 ± 0.0080 m^2^/s vs. 0.0208 ± 0.0060 m^2^/s, *P* = 0.077) between the two subgroups. However, A-vortex behavior was significantly different between the W-HG subgroup and the P-HG subgroups. Type 2 diabetic CKD patients with poorly-controlled hyperglycemia had larger A-vortex area (479.06 ± 146.78 mm^2^ vs. 559.96 ± 159.27 mm^2^, *P* = 0.015) and higher A-vortex circulation (0.0221 ± 0.0058 m^2^/s vs. 0.0275 ± 0.0064 m^2^/s, *P* ˂ 0.001) than the patients with well-controlled hyperglycemia. Interestingly, the stronger trend passed from the A-vortex to the S-vortex in P-HG subgroup. Both S-vortex area (524.21 ± 165.52 mm^2^ vs. 607.87 ± 185.33 mm^2^, *P* = 0.029) and S-vortex circulation (0.0174 ± 0.0072 m^2^/s vs. 0.0213 ± 0.0074 m^2^/s, *P* = 0.015) were increased in the P-HG subgroup compared to the W-HG subgroup. Moreover, the Relative duration of systolic vortex was found be extended in the P-HG subgroup compared to the W-HG subgroup (0.7436 ± 0.0772 vs. 0.7845 ± 0.0752, *P* = 0.014).

### Correlation and risk factors analysis for LV Vortex

In the present study, the correlations among the vortex parameters, between the vortex parameters and the HbA1c or the conventional LV filling parameters in type 2 diabetic patients with early CKD were investigated. There were significant correlations between vortex area and vortex circulation, as observed for the E-vortex (r = 0.580, *P* ˂ 0.001), A-vortex (r = 0.624, *P* ˂ 0.001), and S-vortex (r = 0.660, *P* ˂ 0.001). Relative duration of systolic vortex was weakly associated correlated with S-vortex area (r = 0.211, *P* = 0.048), while significantly associated with A-vortex area (r = 0.293, P = 0.005), A-vortex circulation (r = 0.320, *P* = 0.002) and S-vortex circulation (r = 0.278, *P* = 0.008) in patient group. No significant correlation was found between the Relative duration of diastolic vortex and other vortex parameters.

E-vortex area was associated with E and e′. E-vortex circulation correlated to E and e′. Moreover, A-vortex area was found to be associated with HbA1c, A, and E/A. A-vortex circulation was correlative to HbA1c, A, and E/A. Furthermore, our data showed S-vortex area was associated with HbA1c, A, and E/A. S-vortex circulation had close correlation to HbA1c, A, and E/A. Relative duration of systolic vortex was associated with HbA1c. The results of the Spearman rank correlation test were presented in Table 2.

**Table 2:** Correlation between left ventricular vortex features and filling parameters in type 2 diabetic patients with early CKD

Variables	E		e′		A		E/A		HbA1c	
	r	*P*	r	*P*	r	*P*	r	*P*	r	*P*
E-vortex area	0.300	0.005*	0.370	< 0.001*	0.206	0.051	0.015	0.892	0.104	0.333
E-vortex circulation	0.267	0.011*	0.322	0.002*	0.144	0.179	0.102	0.344	0.079	0.464
A-vortex area	-0.062	0.562	0.198	0.062	0.277	0.009*	-0.268	0.011*	0.322	0.002*
A-vortex circulation	-0.030	0.782	0.037	0.729	0.289	0.006*	-0.214	0.044*	0.509	< 0.001*
S-vortex area	-0.109	0.308	-0.149	0.162	0.302	0.004*	-0.291	0.006*	0.235	0.027*
S-vortex circulation	-0.146	0.173	-0.201	0.059	0.281	0.008*	-0.300	0.004*	0.277	0.009*
RD of systolic vortex	-0.124	0.246	-0.009	0.933	0.018	0.869	-0.078	0.470	0.229	0.031*
RD of diastolic vortex	-0.016	0.880	0.043	0.686	-0.099	0.354	0.053	0.622	-0.070	0.517

E-vortex: vortex during early diastole phase, A-vortex: vortex during atrial contraction phase, S-vortex: vortex during the isovolumic contraction phase, RD: Relative duration

*P < 0.05

### Reproducibility

**Fig. 4:**
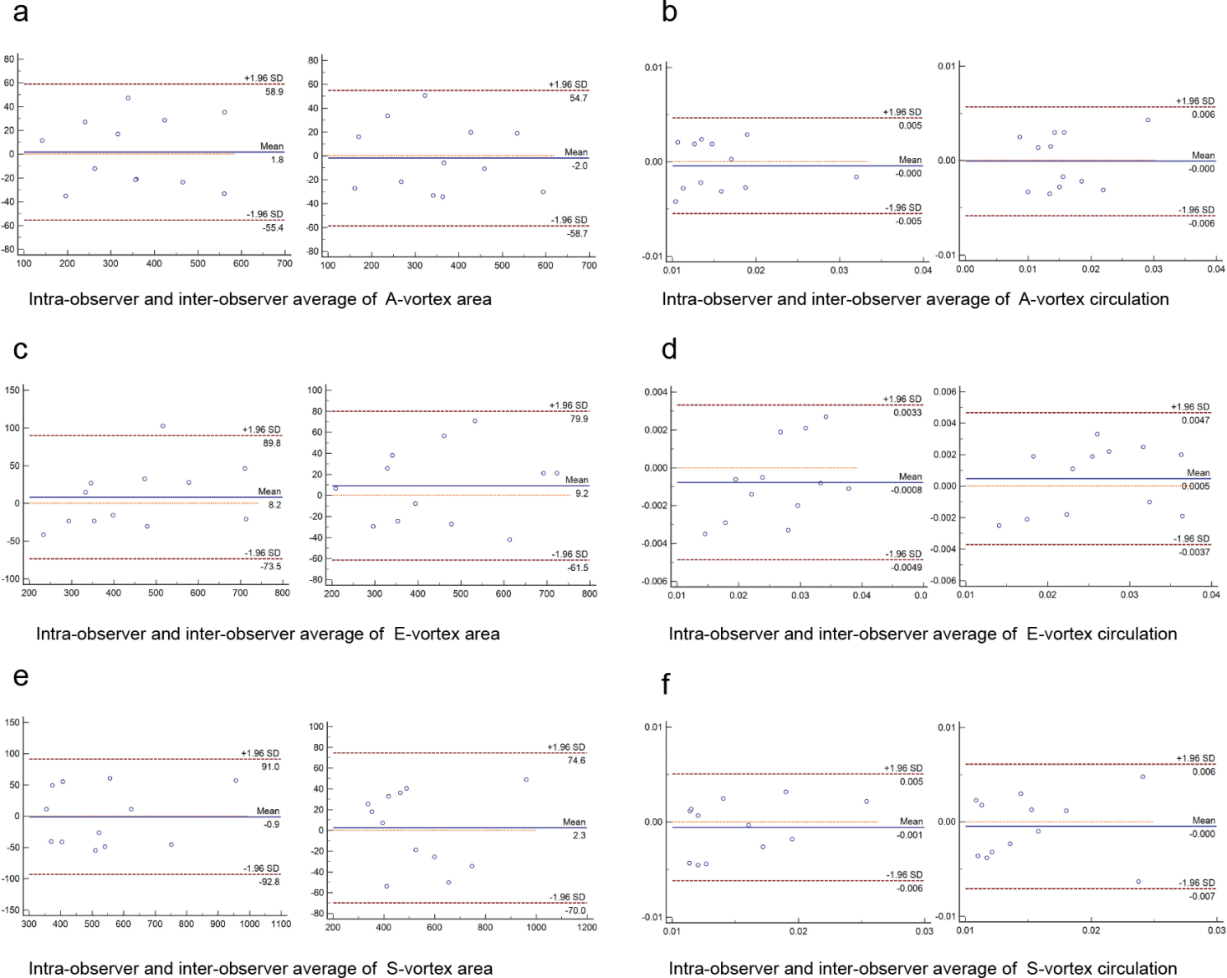
Bland-Altman plots of intra-observer and inter-observer variability. Intra-observer and variability of A-vortex area **(a)**, A-vortex circulation **(b)**, E-vortex area **(c)**, E-vortex circulation **(d)**, S-vortex area **(e)** and S-vortex circulation **(f)**

The intra-observer and inter-observer variabilities for LV vortex values were illustrated in Fig. 4; Table 3. Bland-Altman analysis showed that measurements for E-vortex area, E-vortex circulation A-vortex area, A-vortex circulation, S-vortex area, S-vortex circulation, Relative duration of diastolic vortex and Relative duration of systolic vortex exhibit good reproducibility.

**Table 3:** Reproducibility analysis for left ventricular vortex

		ICC (95%CI)	LOA
E-vortex area	Intra-observer	0.97 (0.89–0.99)	8.2 (−73.5 to 89.8)
E-vortex area	Inter-observer	0.98 (0.92–0.99)	9.2 (−61.5 to 79.9)
E-vortex circulation	Intra-observer	0.96 (0.86–0.98)	−0.0008 (−0.049 to 0.0033)
E-vortex circulation	Inter-observer	0.96 (0.87–0.99)	0.0005 (−0.0037 to 0.0047)
A-vortex area	Intra-observer	0.98 (0.93–0.99)	1.8 (−55.4 to 58.9)
A-vortex area	Inter-observer	0.98 (0.93–0.99)	−2.0 (−58.7 to 54.7)
A-vortex circulation	Intra-observer	0.91 (0.74–0.97)	−0.000 (−0.005 to 0.005)
A-vortex circulation	Inter-observer	0.88 (0.65–0.96)	−0.000 (−0.006 to 0.006)
S-vortex area	Intra-observer	0.97 (0.90–0.99)	−0.9 (−92.8 to 91.0)
S-vortex area	Inter-observer	0.98 (0.94–0.99)	2.3 (−70.0 to 74.6)
S-vortex circulation	Intra-observer	0.82 (0.50–0.94)	−0.001 (−0.006 to 0.005)
S-vortex circulation	Inter-observer	0.78 (0.42–0.93)	−0.000 (−0.007 to 0.006)

CI: confidence interval, ICC: intraclass correlation coefficient, LOA: limits of agreement

## Discussion

Both energy loss and vortex are important parameters based on VFM technology, and helpful in exploring the intracardiac blood flow status and evaluating LV function [31]. The LV energy loss in early CKD had been firstly determined in our previous work [7]. In the current study, we further provided the first evidence on the visualization and quantification of LV vortices using VFM in the early CKD population. Type 2 diabetic patients with early CKD had a small and weak early filling vortex, but a large and intense late filling vortex in the diastolic phase. The late filling vortex became larger and more intense in those patients with poorly controlled hyperglycemia, and grew into a strong systolic vortex, resulting in a long duration of LV vortex in the systolic phase. Using correlation analysis, poorly controlled hyperglycemia was found to contribute to the aberrant vortex behavior across the late diastolic phase and the systolic phase.

Various forms of LV vortex formation convey diagnostic and prognostic information regarding the function of the heart, which is widely applied to assess functional status of the heart in a variety of diseases [11]. Chen et al. conducted an assessment of LV hemodynamics in uremia patients (late stages of CKD) using vortex formation analysis through VFM [24]. The present study builds upon our previous research on LV hemodynamics in early CKD, specifically focusing on investigating the characteristics of LV vortex. A small and weak early diastolic vortex, but a large and intense late diastolic vortex were observed in the type 2 diabetic patients with early CKD. The change of diastolic vortices in the type 2 diabetic patients with early CKD was similar to that in non-CKD patients with diastolic dysfunction. The early diastolic vortex had a small size and weak intensity with no statistical difference, and the late diastolic vortex became significantly stronger in non-CKD patients with diastolic dysfunction [29, 32]. Our data further confirmed that the LV filling parameters were closely associated with the behavior of diastolic vortices. E (reflecting LV early filling) was close related to the size and the intensity of early diastolic vortex, and A (reflecting LV late filling) had a close correlation with the late diastolic vortex characteristics, including the area and circulation. E/A, as a common echocardiographic parameter representing diastolic dysfunction, respectively displayed an inverse correlation to the size and the intensity of the late diastolic vortex behavior. CKD-related increases to myocardial collagen causes diffuse myocardial interstitial fibrosis, resulting in decline of myocardial relaxation [3], which already appears in the early stages of type 2 diabetic CKD [25]. This deterioration of myocardial relaxation further leads to imperceptible diastolic dysfunction, with a decrease in passive filling and a compensatory increase in active atrial filling. Correspondingly, the early diastolic E-vortex decreased in size and intensity, while the late diastolic A-vortex became dominant with an enhanced size and intensity in type 2 diabetic patients with early CKD. A recent high-quality review regarding VFM highlighted that the diastolic vortices redirect blood flow in the outflow tract, reduce friction forces by maintaining blood flow in motion, and then contribute to LV filling [11]. The inverse change of the two diastolic vortices was considered as a positive adaptation for maintaining energetic efficiency of cardiac blood flow and functional compensation of cardiac blood flow for diastolic dysfunction. This functional compensation was more obvious in type 2 diabetic CKD patients with poorly controlled hyperglycemia, manifested by the presence of a stronger A-vortex. Poorly controlled hyperglycemia has been confirmed to exacerbate diastolic dysfunction in patients with diabetes and CKD [33], and this was effectively detected by LV vortex examination, especially if their hearts have a normal morphology shape. It can be inferred that LV vortices have diagnostic and prognostic significance for assessing the functional status of the heart in patients with diabetes and CKD, with great prospects in the clinical application.

During the isovolumic contraction phase, LV pressure increases rapidly while its volume remained unchanged; simultaneously, LV flow altered its direction into the outflow tract and aorta. This physiological mechanism has been known to prevent flow separation and avoid excessive dissipation of energy [30]. The systolic vortex mainly presents across the isovolumic contraction phase and early ejection phase with a suitable size and intensity, and appears to store energy and optimize efficiency for the systolic LV flow [22]. In the current study, the behavior of the systolic vortex displayed a significant change in type 2 diabetic CKD patients with poorly controlled hyperglycemia. The systolic vortex of the P-HG subgroup was almost observed until the end of the ejection phase, and had a large size and high intensity during the isovolumic contraction phase. The long duration of the systolic vortex indicated that the vortex flow never left the left ventricle, which might impede effectively ejection in the patients with poorly controlled hyperglycemia. Fukuda et al. found that the longer the vortex persisted through systole, the more the LV flow energy was lost [19], thus the large area and enhanced circulation of the S-vortex signified energy dissipation of the LV flow. The changed behavior of systolic vortex was consistent with a previous study regarding uremic patients with a definite decline in LV systolic function [24]. Importantly, there were no significant differences in morphological changes and ejection fraction between our early CKD (non-dialysis) patient subgroups, suggesting that systolic hemodynamics abnormalities might appear earlier than the systolic dysfunction. Interestingly, our correlation data showed that HbA1c, A (reflecting LV late filling) and E/A (reflecting LV diastolic function) were closely related to the S-vortex characteristics. Considering that a stronger A-vortex also existed in those patients with poorly controlled hyperglycemia, we supposed that the aberration of S-vortex behavior continued from the stronger of A-vortex, and might be a presentation of relatively severe diastolic dysfunction in systolic blood flow. Additionally, poorly controlled hyperglycemia-induced increase of cardiac afterload played a direct role in the efficiency of blood flow to the heart, which might partly explain the long duration, large size, and high intensity of S-vortex.

Type 2 diabetic kidney disease is the leading cause of CKD worldwide. In view of its widespread prevalence, we chose to focus on the early CKD patients with a diagnostic etiology of type 2 diabetes. In 2020, KDIGO published the first clinical practice guideline directed specifically for clinical management of patients with diabetes and CKD, in which an individualized HbA1C target ranging from < 6.5% to < 8.0% was recommended in patients with diabetes and non-dialysis CKD [2]. Glycemic control had been widely believed the key therapeutic strategy for impeding the CKD progression and avoiding unfortunate cardiovascular events [1]. However, some patients neglect their glycemic condition and do not take proactive steps until they experience an unfortunate cardiac event. Indeed, glycemic control was not well performed in our type 2 diabetic CKD population. Importantly, our data indicated that poorly controlled hyperglycemia is involved in the cardiac hemodynamic abnormalities across the late diastolic and systolic phase in type 2 diabetic patients with early CKD, as visualized by LV vortex using VFM, and found glycemia is independently associated with the features of A-vortex and S-vortex. The correlation of glycemic level and cardiac hemodynamics abnormalities had been confirmed by previous work in diabetic patients by using VFM-enabled EL assessment [34]. Collectively, our data offered the new evidence on the role of glycemia in the LV vortex in the type 2 diabetic CKD patients, which highlights the importance of controlling glycemia for these patients from a hemodynamic perspective. Moreover, sodium/glucose cotransporter 2 (SGLT2) inhibitors, a novel highly efficacious drug targeting glycemic control, have multifaceted benefits in nephrology, cardiology, endocrinology, and primary care, and are expected to further optimize the benefits of glycemic control in patients with diabetes and CKD [35].

This study has several limitations. The vortex posterior component was not analyzed in the present study, as previously explained, its role in cardiac function and hemodynamics seems to be less relevant [13]. Additionally, our present work was a single-center, cross-sectional study with a relatively small sample size. Future studies with larger sample sizes and long-term follow-ups are expected to further confirm the prognostic value of LV vortex in diabetic patients with early CKD. Moreover, we focused only on the impact of hyperglycemia on cardiac vortex in diabetic patients with early CKD. Hyperlipidemia and hypertension, two other common clinical symptoms of diabetic CKD, and need to be investigated in future studies targeting the diagnostic and prognostic significance of cardiac vortex formation in the patients with diabetes and CKD.

## Conclusion

In conclusion, aberrant behaviors of LV vortex were observed in type 2 diabetic patients with early CKD, particularly in those with uncontrolled hyperglycemia. LV vortex may be a practical tool for the convenient identification of LV diastolic dysfunction caused by hyperglycemia in early CKD. This study provided hemodynamic evidence for the strict management of hyperglycemia in type 2 diabetic CKD patients from the cardiac perspective.

### Electronic supplementary material

Below is the link to the electronic supplementary material.

Supplementary Material 1

Supplementary Material 2

## Data Availability

The datasets generated and analyzed in this study are available from the corresponding author on request.

## Abbreviations

A: mitral late filling wave peak velocity
A-vortex: vortex during atrial contraction phase
CKD: chronic kidney disease
DM: diabetes mellitus
E: mitral early filling wave peak velocity
E-vortex: vortex during early diastole phase
e’: early mitral annular peak velocity on septal side
eGFR: estimated glomerular filtration rate
HbA: glycosylated hemoglobin
LVEDD/SD: left ventricular end-diastolic/systolic diameter
LVEDV/SV: left ventricular end-diastolic/systolic volume
LVEF: left ventricular ejection fraction
P-HG: patients with poorly- controlled hyperglycemia
S-vortex: vortex during the isovolumic contraction phase
W-HG: patient with well-controlled hyperglycemia

## References

[1] Doshi SM, Friedman AN (2017). Diagnosis and management of type 2 Diabetic kidney disease. Clin J Am Soc Nephrol.

[2] Mottl AK, Alicic R, Argyropoulos C, Brosius FC, Mauer M, Molitch M (2022). KDOQI US Commentary on the KDIGO 2020 Clinical Practice Guideline for Diabetes Management in CKD. Am J Kidney Dis.

[3] Wu PY, Huang JC, Chen SC, Chen LI (2018). Type 2 diabetes mellitus-related changes in left ventricular structure and function in patients with chronic kidney disease. Oncotarget.

[4] Zhang Y, Wang J, Ren Y, Yan WF, Jiang L, Li Y (2021). The additive effects of kidney dysfunction on left ventricular function and strain in type 2 diabetes mellitus patients verified by cardiac magnetic resonance imaging. Cardiovasc Diabetol.

[5] Ng KP, Jain P, Gill PS, Heer G, Townend JN, Freemantle N (2016). Results and lessons from the spironolactone to prevent Cardiovascular events in early stage chronic kidney disease (STOP-CKD) randomised controlled trial. BMJ Open.

[6] Jagieła J, Bartnicki P, Rysz J (2020). Selected cardiovascular risk factors in early stages of chronic kidney disease. Int Urol Nephrol.

[7] Chen X, Wang Y, Wang W, Yuan L, Qi Z, Song D (2020). Assessment of left ventricular energy loss using vector flow mapping in patients with stages 1–3 chronic kidney disease. BMC Cardiovasc Disord.

[8] Pluta A, Stróżecki P, Krintus M, Odrowąż-Sypniewska G, Manitius J (2015). Left ventricular remodeling and arterial remodeling in patients with chronic kidney disease stage 1–3. Ren Fail.

[9] Rodriguez Muñoz D, Markl M, Moya Mur JL, Barker A, Fernández-Golfín C, Lancellotti P (2013). Intracardiac flow visualization: current status and future directions. Eur Heart J Cardiovasc Imaging.

[10] Kim IC, Hong GR (2019). Intraventricular Flow: more than pretty Pictures. Heart Fail Clin.

[11] Kheradvar A, Rickers C, Morisawa D, Kim M, Hong GR, Pedrizzetti G (2019). Diagnostic and prognostic significance of cardiovascular vortex formation. J Cardiol.

[12] Hong GR, Kim M, Pedrizzetti G, Vannan MA (2013). Current clinical application of intracardiac flow analysis using echocardiography. J Cardiovasc Ultrasound.

[13] Rodríguez Muñoz D, Moya Mur JL, Fernández-Golfín C, Becker Filho DC, González Gómez A, Fernández Santos S (2015). Left ventricular vortices as observed by vector flow mapping: main determinants and their relation to left ventricular filling. Echocardiography.

[14] Avesani M, Degrelle B, Di Salvo G, Thambo JB, Iriart X (2021). Vector flow mapping: a review from theory to practice. Echocardiography.

[15] Gharib M, Rambod E, Kheradvar A, Sahn DJ, Dabiri JO (2006). Optimal vortex formation as an index of cardiac health. Proc Natl Acad Sci U S A.

[16] Kheradvar A, Assadi R, Falahatpisheh A, Sengupta PP (2012). Assessment of transmitral vortex formation in patients with diastolic dysfunction. J Am Soc Echocardiogr.

[17] Hong GR, Pedrizzetti G, Tonti G, Li P, Wei Z, Kim JK (2008). Characterization and quantification of vortex flow in the human left ventricle by contrast echocardiography using vector particle image velocimetry. JACC Cardiovasc Imaging.

[18] Goya S, Wada T, Shimada K, Hirao D, Tanaka R (2018). The relationship between systolic vector flow mapping parameters and left ventricular cardiac function in healthy dogs. Heart Vessels.

[19] Fukuda N, Itatani K, Kimura K, Ebihara A, Negishi K, Uno K et al. Prolonged vortex formation during the ejection period in the left ventricle with low ejection fraction: a study by vector flow mapping. J Med Ultrason (2001). 2014;41(3):301 – 10. 10.1007/s10396-014-0530-3 27277903

[20] Cimino S, Palombizio D, Cicogna F, Cantisani D, Reali M, Filomena D (2017). Significant increase of flow kinetic energy in nonresponders patients to cardiac resynchronization therapy. Echocardiography.

[21] Son JW, Park WJ, Choi JH, Houle H, Vannan MA, Hong GR (2012). Abnormal left ventricular vortex flow patterns in association with left ventricular apical thrombus formation in patients with anterior myocardial infarction: a quantitative analysis by contrast echocardiography. Circ J.

[22] Han Y, Huang L, Li Z, Ma N, Li Q, Li Y (2019). Relationship between left ventricular isovolumic relaxation flow patterns and mitral inflow patterns studied by using vector flow mapping. Sci Rep.

[23] Sherrid MV, Kushner J, Yang G, Ro R (2017). Mitral valve coaptation and its relationship to late diastolic flow: a color Doppler and vector flow map echocardiographic study in normal subjects. Echocardiography.

[24] Chen R, Zhao BW, Wang B, Tang HL, Li P, Pan M (2012). Assessment of left ventricular hemodynamics and function of patients with uremia by vortex formation using vector flow mapping. Echocardiography.

[25] Abdelaaty T, Morsy E, Rizk M, Shokry A, Abdelhameid A, Fathalla R (2022). Relation of serum heart type fatty acid binding protein to left ventricular diastolic dysfunction in patients with type 2 diabetes and early diabetic kidney disease. J Diabetes Complications.

[26] Lentini P, Zanoli L, Ronco C, Benedetti C, Previti A, Laudadio G et al. The vascular disease of diabetic kidney disease. Cardiorenal Med. 2022. 10.1159/000527274 36279858

[27] Mitchell C, Rahko PS, Blauwet LA, Canaday B, Finstuen JA, Foster MC (2019). Guidelines for performing a comprehensive transthoracic echocardiographic examination in adults: recommendations from the American Society of Echocardiography. J Am Soc Echocardiogr.

[28] Chan JSK, Lau DHH, Fan Y, Lee AP. Age-Related Changes in Left Ventricular Vortex Formation and Flow Energetics. J Clin Med. 2021;10(16). 10.3390/jcm10163619 PMC8397127 34441914

[29] Zhou BY, Xie MX, Wang J, Wang XF, Lv Q, Liu MW (2017). Relationship between the abnormal diastolic vortex structure and impaired left ventricle filling in patients with hyperthyroidism. Med (Baltim).

[30] Akiyama K, Maeda S, Matsuyama T, Kainuma A, Ishii M, Naito Y (2017). Vector flow mapping analysis of left ventricular energetic performance in healthy adult volunteers. BMC Cardiovasc Disord.

[31] Wang W, Wang Y, Chen X, Yuan L, Bai H (2019). Evaluation of left ventricular diastolic function based on flow energetic parameters in chronic kidney disease with diastolic dysfunction. Echocardiography.

[32] Wang Y, Hong J, Yu R, Xu D (2021). Evaluation of left ventricular function by vector flow mapping in females with systemic lupus erythematosus. Clin Rheumatol.

[33] Cooper ME (2004). Importance of advanced glycation end products in diabetes-associated cardiovascular and renal disease. Am J Hypertens.

[34] Li CM, Bai WJ, Liu YT, Tang H, Rao L (2017). Dissipative energy loss within the left ventricle detected by vector flow mapping in diabetic patients with controlled and uncontrolled blood glucose levels. Int J Cardiovasc Imaging.

[35] DeFronzo RA, Reeves WB, Awad AS (2021). Pathophysiology of diabetic kidney disease: impact of SGLT2 inhibitors. Nat Rev Nephrol.

