# Supplementary material for: Vector flow mapping analysis of left ventricular vortex performance in type 2 diabetic patients with early chronic kidney disease
Source: BMC Cardiovasc Disord. 2023 Sep 1;23:434. doi: 10.1186/s12872-023-03474-7 (PMC10474629; doi:10.1186/s12872-023-03474-7)
Supplement: Supplementary file 1 — Supplementary Material 1 [file 12872_2023_3474_MOESM1_ESM.docx]

More vector flow maps were provided in Figure. S. Representative images of E-vortex, A-vortex and S-vortex were selected from a healthy control, a patient in W-HG subgroup, and a patient in P-HG subgroup. Patients had a small and weak E-vortex, but a large and intense A-vortex compared to controls. Patient in the P-HG subgroup had stronger A-vortex and S-vortex than the patient in W-HG subgroup.
