# Supplementary figures and images for: Vector flow mapping analysis of left ventricular vortex performance in type 2 diabetic patients with early chronic kidney disease
Source: BMC Cardiovasc Disord. 2023 Sep 1;23:434. doi: 10.1186/s12872-023-03474-7 (PMC10474629; doi:10.1186/s12872-023-03474-7)

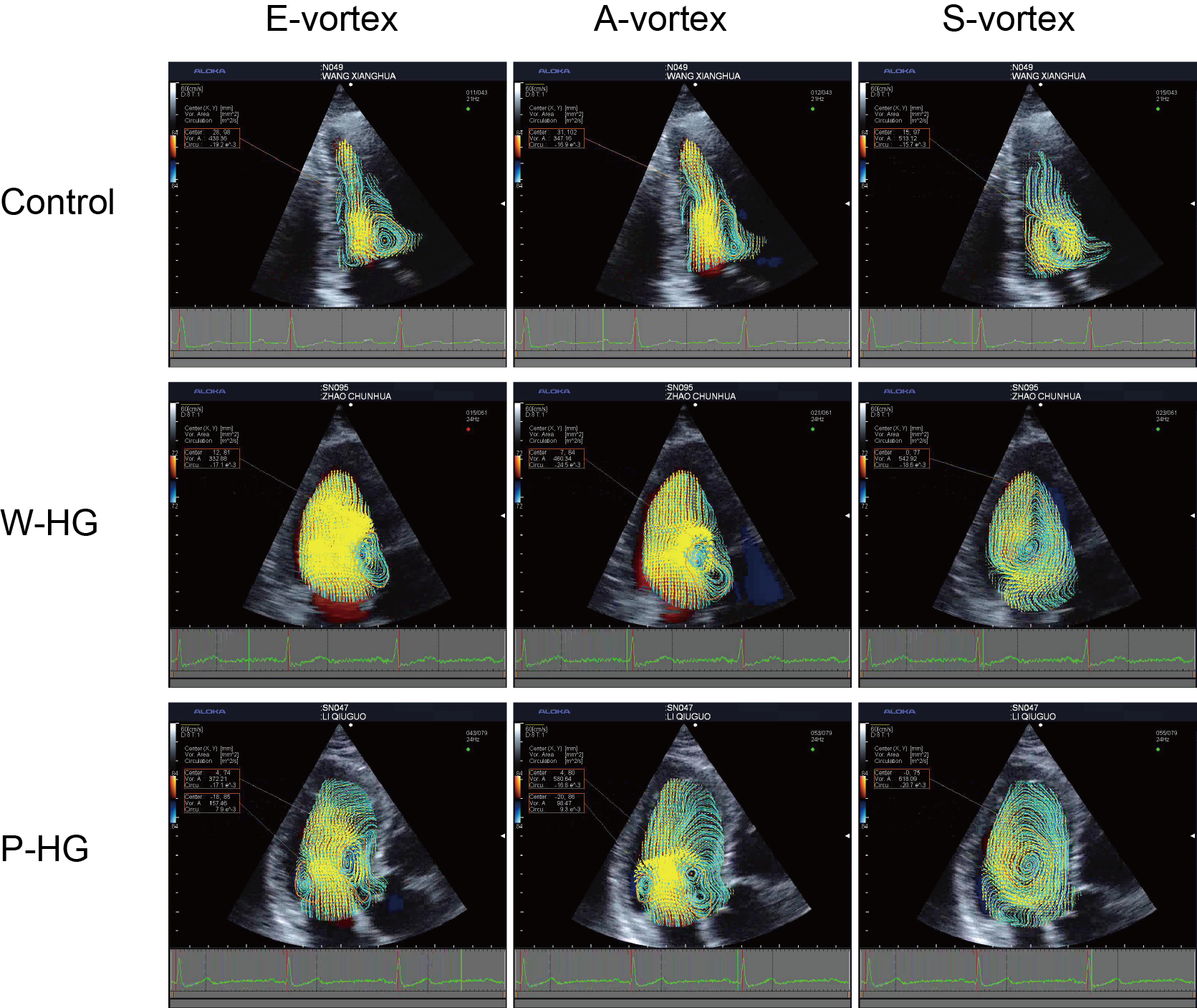

Supplement: Supplementary file 2 — Supplementary Material 2 [file 12872_2023_3474_MOESM2_ESM.tif]
